# Cheapest Food

**Published:** 1877-07

**Authors:** 


### CHEAPEST FOOD,

And among the most nutritious that can be eaten, is *White Beans;* cheapest to the consumer, and more profitable to the producer than wheat at one dollar and a quarter a bushel, when the beans sell at one dollar a bushel. But this last winter they have readily sold at three dollars a bushel. The amount of nutriment in white beans is ninety-three per cent; so that one dollar's worth of white beans, at two dollars a bushel, affords double the nutriment of a dollar's worth of potatoes, at a dollar a bushel. White beans, at two dollars a bushel, are a cheaper food than potatoes at half a dollar a bushel—beans having no waste.

